# The “Decline and Fall Off”

**Published:** 1891-06

**Authors:** 


[Not of the "Russian Empire," but of the Texas State Medical Association.]

A Texas journal not very kindly disposed towards the Texas State Medical Association, says: "The association is losing its best and most useful members, and its number is rapidly diminishing. What is to be done?"

The extent of this great decadence can best be appreciated in light of the record:

In 1884 the roll numbered........................280 members
In 1891 the roll numbers..........................482 "
showing a *net* gain of 202 members; 72 per cent; or an annual average of twenty-five new members.

"Under the influence of spirited journalism" (New York *Medical Record* referring to the work of DANIEL'S TEXAS MEDICAL JOURNAL in promoting organization), four hundred and forty-nine members have been added to the roll since 1884, a gain of 160 per cent., or yearly average of 56 new members; as follows:

| | | | |
|---|---|---|---|
| At Belton, in 1884, there were | admitted | | 89 |
| At Houston, in 1885 | " | " | 39 |
| At Dallas, in 1886 | " | " | 87 |
| At Austin, in 1887 | " | " | 52 |
| At Galveston, in 1888 | " | " | 32 |
| At San Antonio, in 1889 | " | " | 58 |
| At Fort Worth, in 1890 | " | " | 48 |
| At Waco, in 1891 | " | " | 44 |
| Total | | | 449 |

In the eight years over one hundred and eighty have been dropped from the roll for non-payment of dues three consecutive years (not "the best and most useful members," surely); over fifty have died; a number have removed from the State, *four* have resigned, and five have been expelled. By which of these avenues, then, has the association "lost its best and most useful members?" and "what *is* to be done," really?

